# Practical Instruction in Microscopy

**Published:** 1852-01

**Authors:** 


*Practical Instruction in Microscopy.*—We are happy to announce that, in compliance with the wishes of several of the practitioners of this city, Dr. John C. Dalton, Prof. of Physiology in the Medical Department of the University of Buffalo, proposes to give a series of lectures on Microscopy, designed especially for physicians desirous of becoming practically conversant with the more important and interesting facts belonging in this newly developed province of medical science. The course will embrace about twelve lectures, to be given in some convenient central place not yet designated. It is proposed that the lectures shall commence immediately after the present lecture session in the Medical College, which will be about the first of March next, and continue three or four weeks, three or four lectures being given weekly.

The subjects to be treated of in the course will be arranged as follows:

The first lecture will be occupied with a description of the microscope, the different varieties of the instrument, the mode of using it, manipulations, test objects, &c.

The different healthy tissues of the body will then be taken up in detail, and their microscopic structure demonstrated; together with the blood and circulation, the ovum, and the development of the tissues.

The chrystalline deposits and other microscopic appearances to be met with in the urine will then be considered, as well as those occurring in other fluid secretions.

Finally, the latter part of the course will embrace the minute structure of the different morbid products, solid and fluid; pus, false membrane, tubercle, cancer, fibrous and fatty tumors, epithelial growths, &c. &c.

In treating the foregoing subjects, the object will be to render the study practical; that is to say, demonstrative. For this end several microscopes will be employed, and the class subdivided into sections, the lectures and illustrations being repeated for each section. In this way ample opportunity will be offered for every member of the class to become practically familiar with the microscopical appearances embraced in the course.

It is hardly necessary to say that developments by means of the microscope have, within a few years, given a new aspect to the study of anatomy, physiology, and pathology. Facts disclosed by this method of investigation have not only rendered these branches of medical science more attractive, but have added, in no small measure, to the extent and importance of their practical relations. While we can scarcely imagine any pursuit to possess greater scientific interest, some knowledge of microscopy has become quite indispensable to the accomplished practitioner. To prosecute the pursuit with success, however, requires tact in the use of the microscope, preparing objects, etc., which few practitioners have time and facilities to acquire without assistance from those who have bestowed particular attention upon this branch of science. Hence, a course of practical or demonstrative lectures is almost necessary for the novitiate. Such a course may be expected to answer all the useful purposes pertaining to the practice of medicine, and to supply preliminary attainments to those who wish to prosecute farther investigations. Dr. Dalton, as most of our readers are doubtless aware, has devoted himself with great zeal and success to scientific pursuits involving practical acquaintance with the microscope. For ourselves, the contemplated course of lectures offers an opportunity which we should be very reluctant to forego.

Particulars respecting the days and hours of the lectures, the place where they will be given, etc. will be announced in the Journal for the next month.

As it is desirable that the size of the class should be ascertained, in order to arrange the subdivisions into sections, persons who desire to attend are requested to signify their intention at an early date.

The fee for the course will be but five dollars.

